# Supplementary material for: A comprehensive biomechanical analysis of the barbell hip thrust
Source: PLoS One. 2021 Mar 29;16(3):e0249307. doi: 10.1371/journal.pone.0249307 (PMC8006986; doi:10.1371/journal.pone.0249307)
Supplement: Attachment — Submitted filename: PONE-D-20-27535_reviewer.pdf [file pone.0249307.s002.pdf]

PONE-D-20-27535_reviewer

General comments

The focus of the study is the biomechanics of the barbell hip thrust exercise and a research article that contributes to practice. However, there are a few general comments to be completed. Firstly, a free body diagram might be added to the materials and methods section. In addition, a figure can be used showing the experimental setup. Secondly, Newton-Euler inverse dynamic formulas might be presented in the method section.

Detailed comments about this study are below.

Abstract

The results of the study are presented clearly in abstract section.

Introduction

The reasons for studying the biomechanics of hip thrust exercise are presented in the introduction section. However, the hypothesis of the study has been not included in the introduction section. What is the hypothesis of the study? Please add hypothesis to the introduction.

Methods

Please, add a free body diagram to the materials and methods section. A considerable simplification can be made to understand the kinetic and kinematic dimensions of the hip thrust movement if the forces and moments in the pelvic-trunk joint and lower extremity joints are defined by the free body diagram. In addition, a figure that shows the marker set attached to the body can be added to method section, or a figure showing the experimental setup. As known, Euler’s 3D equations of motion are used for kinetic analyses of a segment Please, add to the text Newton-Euler inverse dynamic procedures used for calculation of moment. On the other hand, Euler's equations also require data such as angular acceleration and moments of inertia of lower extremity segments (thigh, shank and foot). It can be mentioned in the text if angular acceleration and moments of inertia of the lower limb segments data were used in calculation.

Results

External kinematics and kinetics and joint kinematics and kinetics were presented clearly in Table1, Table2, and Table3, and were supported with the figures.

Discussion

It was reported in the study that the hip extensor moment decreased during the lift contrary to the literatüre. This is the most important result of the study. The main results of the study, which contradicts with the literature, are very well discussed with fine details. I applaud the authors.

Page 14, line 284

Both dot and comma were used together in the end of the sentence. Please, remove the comma.

Page 14, line 303

Please, add a dot at the end of the sentence.
