# Supplementary material for: A comprehensive biomechanical analysis of the barbell hip thrust
Source: PLoS One. 2021 Mar 29;16(3):e0249307. doi: 10.1371/journal.pone.0249307 (PMC8006986; doi:10.1371/journal.pone.0249307)
Supplement: Attachment — Submitted filename: Response to Reviewers.docx [file pone.0249307.s004.docx]

**Journal Requirements.**

Please review your reference list to ensure that it is complete and correct. If you have cited papers that have been retracted, please include the rationale for doing so in the manuscript text or remove these references and replace them with relevant current references. Any changes to the reference list should be mentioned in the rebuttal letter that accompanies your revised manuscript. If you need to cite a retracted article, indicate the article’s retracted status in the References list and also include a citation and full reference for the retraction notice.

*Thank you for this comment. From the original submission there have been no references removed from the manuscript. Following the first revision, three references were added to the manuscript (23, 25, 26) to address reviewer comments, which resulted in changes to the numerical order of the in-text citations. The first revision of the manuscript highlighted the changes to the numerical order of in-text citations, but this did not reflect a removal/ change in references used. Since the original submission, reference 8 had been allocated a volume and page number, which was updated in the first revision of the manuscript.*

**Reviewer(s)’ Comments to the Author:**

**Specific comments**

Figure 2. How did you handle the moment acting between the toe and the ground (I guess authors assumed that moment to be zero)? The details of the method should be clearly stated.

- *In the current analysis, the foot was modelled as a single rigid segment with the distal endpoint at the metatarsophalangeal joint (MTPJ). The moment between the distal end of the foot and ground was not included in our analysis and was assumed to be zero. As the foot was modelled as a single segment, the joint moment acting at the MTPJ (between foot and toe) was also not considered in our analysis, which is consistent in the resistance training literature. Whilst the MTPJ has been shown to generate and absorb energy in locomotive tasks (e.g. sprinting), evidence has suggested (e.g. Bezodis et al., 2012. DOI: https://doi.org/10.1123/jab.28.2.222) that if the specific focus of the study is the kinetics of the ankle, knee and hip, the current method is appropriate.*

*In the revised manuscript, the following sentence has been included to the methods section for clarity (line 151-154):*

*“The distal end of the foot was defined at the metatarsophalangeal joint, and the moment acting between the distal end of the foot and the ground was assumed to be zero.”*
